# Serotonin and Corticosterone Rhythms in Mice Exposed to Cigarette Smoke and in Patients with COPD: Implication for COPD-Associated Neuropathogenesis

**DOI:** 10.1371/journal.pone.0087999

**Published:** 2014-02-10

**Authors:** Isaac K. Sundar, Hongwei Yao, Yadi Huang, Elizabeth Lyda, Patricia J. Sime, Michael T. Sellix, Irfan Rahman

**Affiliations:** 1 Department of Environmental Medicine, Lung Biology and Disease Program, University of Rochester Medical Center, Rochester, New York, United States of America; 2 Department of Medicine (Pulmonary), University of Rochester Medical Center, Rochester, New York, United States of America; 3 Department of Medicine, Division of Endocrinology and Metabolism, University of Rochester Medical Center, Rochester, New York, United States of America; University of Texas Medical Branch, United States of America

## Abstract

The circadian timing system controls daily rhythms of physiology and behavior, and disruption of clock function can trigger stressful life events. Daily exposure to cigarette smoke (CS) can lead to alteration in diverse biological and physiological processes. Smoking is associated with mood disorders, including depression and anxiety. Patients with chronic obstructive pulmonary disease (COPD) have abnormal circadian rhythms, reflected by daily changes in respiratory symptoms and lung function. Corticosterone (CORT) is an adrenal steroid that plays a considerable role in stress and anti-inflammatory responses. Serotonin (5-hydroxytryptamine; 5HT) is a neurohormone, which plays a role in sleep/wake regulation and affective disorders. Secretion of stress hormones (CORT and 5HT) is under the control of the circadian clock in the suprachiasmatic nucleus. Since smoking is a contributing factor in the development of COPD, we hypothesize that CS can affect circadian rhythms of CORT and 5HT secretion leading to sleep and mood disorders in smokers and patients with COPD. We measured the daily rhythms of plasma CORT and 5HT in mice following acute (3 d), sub-chronic (10 d) or chronic (6 mo) CS exposure and in plasma from non-smokers, smokers and patients with COPD. Acute and chronic CS exposure affected both the timing (peak phase) and amplitude of the daily rhythm of plasma CORT and 5HT in mice. Acute CS appeared to have subtle time-dependent effects on CORT levels but more pronounced effects on 5HT. As compared with CORT, plasma 5HT was slightly elevated in smokers but was reduced in patients with COPD. Thus, the effects of CS on plasma 5HT were consistent between mice and patients with COPD. Together, these data reveal a significant impact of CS exposure on rhythms of stress hormone secretion and subsequent detrimental effects on cognitive function, depression-like behavior, mood/anxiety and sleep quality in smokers and patients with COPD.

## Introduction

Chronic obstructive pulmonary disease (COPD) is the fourth most common cause of death in the developed world, and cigarette smoke (CS) is the major risk factor [1], [2]. COPD is a disabling condition associated with progressive breathlessness and a severe decline in lung function [3]. As COPD progresses, patients develop more frequent and severe exacerbation induced by infections, cigarette smoke and air pollutants, with an increased rate of emergency room visits and hospitalization, mostly at night and in the early morning hours [4]–[6]. In healthy individuals, pulmonary function exhibits a daily rhythm with a noon maximum (12:00 h) and an early morning minimum (04:00 h). The early morning rise in lung function is accompanied with exacerbations of COPD in susceptible individuals [6]. More pronounced nightly drops in forced vital capacity (FVC), forced expiratory volume in one second (FEV_1_) and peak expiratory flow (PEF) are found in smokers than in non-smokers [7]. This response may be due to CS-mediated effects on daily rhythms of stress hormone release, surfactant protein expression, mucus retention/secretion, and lung inflammation that further amplifies the daily rhythm in lung function [6], [8], [9]. Nevertheless, the underlying molecular mechanisms for CS-induced circadian abnormalities are not fully understood.

Sleep abnormalities including symptoms of insomnia, excessive daytime sleepiness, and nocturnal oxygen desaturation are common in patients with COPD [10]–[12]. Disrupted sleep in patients with COPD correlates with respiratory symptoms (cough, sputum production, wheezing), nocturnal oxygen desaturation, hypercapnia, and daily changes in airway caliber and resistance [13]. In addition to primary deficits in lung function and sleep quality; studies reveal an increased rate of depression and anxiety among patients with COPD [14], [15]. Smoking itself is highly associated with various neuropsychiatric disorders [16], and depression often lasts even after smoking cessation [17]. This suggests that neurophysiological function is disrupted in a time-dependent manner in response to CS and in patients with COPD.

Serotonin (5-hydroxytryptamine; 5HT) is a potent neurotransmitter whose levels in the central nervous system are closely associated with mood disorders including depression [18]. Circulating 5HT is derived from both entero-chromaffin cells in the gut, pulmonary neuroendocrine cells and neurons of the Raphe nuclei of the brain [19]. Altered 5HT levels are associated with both CS-related diseases and depression [20], [21]. Genome-wide analyses reveal that 5HT receptor 4 loci, a member of the larger 5HT receptor family is associated with regulation of pulmonary function [22]. Moreover, the level of 5-hydroxyindoleacetic acid (5-HIAA), a primary metabolite of 5HT, is known to correlate with severity of depressive mood disorder in COPD patients [23], [24].

Corticosterone (CORT) is an adrenal steroid and a major component of the stress response [18]. It has been reported that increased steroid hormone levels (including CORT) are associated with both passive and active smoking [25]. In rats, plasma CORT levels are elevated after smoke exposure [26]. Further, nicotine, one of the chemical components of tobacco smoke, can stimulate CORT secretion in mice [27]. In addition, CORT can affect depression-like behavior in rats [28]. Secretion of both 5HT and CORT is under direct and indirect control of the circadian clock in the suprachiasmatic nucleus (SCN). Numerous studies confirm circadian rhythms of circulating CORT through the temporal control of the hypothalamic-pituitary-adrenal (HPA) axis by the SCN [29]–[33]. The interaction between the circadian timing system and serotonergic signaling in the brain is also well established [18], [34], [35]. The amplitude and timing of both CORT and 5HT secretion are altered by stress, and these effects are associated with anxiety and depression, maladies commonly observed in patients with COPD [17], [18], [30], [36]–[38].

Cigarette smoking is a contributing factor in the development of COPD, and smoking may cause rhythmic changes in both CORT and 5HT levels [1], [25]. However, it remains to be determined if smoking and COPD alter circadian rhythms of CORT and 5HT secretion. Here, we measured daily rhythms of plasma CORT and 5HT in mice following acute (3 d), sub-chronic (10 d) and chronic (6 mo) CS exposures. Our aim was to determine if smoking changes the amplitude or timing of these hormones, and to relate these effects to the pathophysiology of COPD. To ascertain the relationship between smoking, COPD and hormone secretory rhythms, we have also quantified plasma CORT and 5HT levels in non-smokers, smokers and patients with COPD.

## Materials and Methods

### Ethics statements

All experimental protocols were performed in accordance with the standards established by the United States Animal Welfare Act, as set forth by the National Institutes of Health guidelines. The research protocol for these studies was approved by the University of Rochester Committee on Animal Research (UCAR).

Subjects/patients recruitment and the study described here were approved by the ethical institutional review board (IRB)/RSRB committee of the University of Rochester Medical Center (#RSRB00028789). All subjects/patients gave their written informed consent.

### Animals

Male C57BL/6J mice purchased from the Jackson Laboratory (Bar Harbor, ME) were housed under a 12∶12 light-dark (LD) cycle with lights on at 6 am, and provided regular chow and water *ad lib*. For sub-chronic (10 days) CS exposure, mice were entrained for a minimum of 2 weeks to an inverted 12∶12 L:D cycle (lights on 6 pm–6 am) prior to CS exposure. For acute (3 days) and chronic (6 mo) CS exposures, mice were housed in a standard 12∶12 L:D cycle with lights on from 6 am–6 pm throughout the experiment.

### Cigarette smoke exposure

Eight week-old mice were used for CS exposure as described previously [39]. We used acute (3 d) or sub-chronic (10 d; [which both induce lung inflammatory responses]) and chronic (6 mo.) [which causes pulmonary emphysema] CS exposure models to determine the effect of acute inflammatory responses and emphysema on daily rhythms of 5HT and CORT secretion. Mice in the 3 d (acute) model were exposed to CS using a Baumgartner-Jaeger CSM2082i cigarette smoking machine (CH Technologies, Westwood, NJ). Mice in the sub-chronic (10 d) and chronic (6 mo.) models were exposed to CS using a Teague TE-10 smoking machine (Teague Enterprises, Davis, CA). All exposures were carried out in the Inhalation Facility at the University of Rochester Medical Center. For acute CS exposure (3 d), mice were placed in individual compartments of a wire cage, which was then placed inside a closed plastic box connected to the smoke source. The smoke was generated from 3R4F research cigarettes containing 11.0 mg of total particulate matter (TPM), 9.4 mg of tar and 0.73 mg of nicotine per cigarette (University of Kentucky, Lexington, KY). The total particulate matter (TPM) in per cubic meter of air in exposure chamber was monitored in real-time with a MicroDust Pro-aerosol monitor (Casella CEL, Bedford, UK), and verified daily by gravimetric sampling [40]. The smoke concentration was set at a value of ∼300 mg/m^3^ TPM by adjusting the flow rate of the diluted medical air, and the level of carbon monoxide in the chamber was 350 ppm [40]. Mice received two 1 h exposures per day (1 h apart), daily for the duration of treatment according to the Federal Trade Commission protocol (1 puff/min of 2 second duration and 35 ml volume) and were sacrificed at 24 h post-last exposure [41]. Control mice were exposed to filtered air in an identical chamber according to the same protocol described for CS exposure. For sub-chronic (10 d) and chronic (6 mo) CS exposures, 3R4F cigarettes were used to generate a mixture of sidestream smoke (89%) and mainstream smoke (11%) at a concentration of ∼100 mg/m^3^ TPM, so as to avoid the possible toxicity to mice at a high concentration of long-term CS exposure [42]. Each smoldering cigarette was puffed for 2 seconds, once every minute for a total of 5 puffs, at a flow rate of 1.05 L/min, to provide a standard puff of 35 cm^3^. Mice received 5 h exposures per day, 5 days/week for consecutive 10 d/6 month, and were sacrificed at 6 h interval after 24 h post-last CS exposure as described previously [39].

### Collection of human samples (non-smokers, smokers, and patients with COPD)

The clinical characteristics of the patients included in the study are presented in Table 1. Blood samples (50–100 ml) were taken from non-smokers, smokers, and patients with COPD as described earlier [43] in between 8 am–2 pm. Plasma was obtained by centrifugation (250×*g*) of lithium-heparinized blood, and assayed for serotonin and corticosterone as described below.

**Table 1:** General clinical characteristics of non-smokers, smokers and COPD patients.

Parameters	Non-smokers	Smokers	COPD
Number of subjects	12	12	11
M:F ratio	6∶6	6∶6	6∶5
Male/Female (%)	(50%/50%)	(50%/50%)	(45%/55%)
Age, yr	61(49–74)	58.8(45–79)	64.1(51–73)
Smoking, pack years	-	38.1(9–70)	52.9(8–120)
Smoking status (Current/Ex-smokers)	-	Current smokers (8)	Current smokers (5)
	-	Ex-smokers (4)	Ex-smokers (6)
FEV_1_ % predicted	105 (89–128)	92 (72–126)	53 (38–104)
ICS; n (%)	-	n = 1 (8.3%)	n = 6 (54.5%)

M:F: Male to Female ratio; FEV_1_: Forced Expiratory Volume in one second; ICS: inhaled corticosteroids.

### Measurements of plasma corticosterone (CORT) and serotonin (5HT) levels

Blood was collected by cardiac puncture. To perform cardiac puncture, mice were anesthetized by sodium pentobarbital (50 mg/kg, intraperitoneally), then the right ventricle was accessed with a 23 gauge needle and 400–500 µl blood was aspirated into a heparin-coated 3 ml syringe. Blood was immediately collected into a 2 ml tube and centrifuged for plasma collection. Plasma was stored at −80°C until further analysis. Plasma corticosterone and serotonin levels were measured using commercially available enzyme-linked immunoassay (EIA) kits (Enzo Life Sciences, Plymouth Meeting, USA) according to the manufacturer's instructions.

## Results

### CS exposure differentially affects daily rhythms of plasma CORT and 5HT

**Figure 1:**
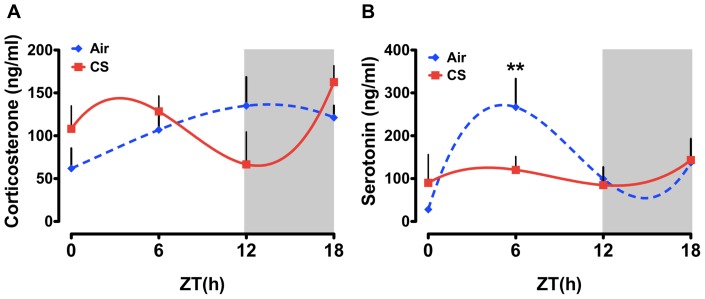
Daily rhythms of plasma CORT and 5HT in mice exposed to acute (3 day) air or CS. Mice were exposed to acute CS during the light phase for 3 days, and plasma samples were collected at every 6-h interval (ZT0, ZT6, ZT12, and ZT18) beginning 24 h after the last exposure. Plasma levels of (A) CORT and (B) 5HT were determined by ELISA. Gray shading indicates the subjective dark phase (ZT12 to ZT24). Data were fit with non-linear regression (multi-order polynomial) analyses. Data are shown as mean ± SEM (n = 3–7 mice per group). **P<0.01 significant compared to CS-exposed mice at ZT6.

It has been shown that plasma serotonin and cortisol levels are directly associated with depression and decreased locomotor activity [17], [18], [24], [36]–[38], [44], and may also be associated with depression in smokers and patients with COPD [45]–[49]. We have recently reported that acute (3 d), sub-chronic (10 d) and chronic (6 mo) CS exposures resulted in a significant decline in the level of locomotor activity and altered the timing of the circadian clock in brain and lung tissues [39]. The effects of CS on nighttime activity were particularly robust and suggest the development of “depression-like” behavior in mice following CS exposure [39]. Here we determined whether our previously reported effects of CS on locomotor activity and molecular clock function [39] correlate with altered levels of circulating 5HT and CORT. As expected, we observed a peak of plasma CORT near lights off (ZT 9.20±3.07) in mice exposed to clean air (controls; Fig 1A, open symbols and Table 2). Following acute CS exposure, we observed an apparent advance of this diurnal pattern of plasma CORT secretion such that the peak occurred at ZT4.13±3.41. The pattern was also marked by a decline in plasma CORT at ZT12 and a subsequent rebound of plasma CORT during the middle of the dark phase (ZT18; Fig. 1A). We next determined the effects of acute CS exposure on plasma 5HT levels in mice. In air-exposed mice, we detected a diurnal rhythm of plasma 5HT marked by a rise to peak levels (Mean ± SD) from ZT0 to ZT7.00±2.26 followed a return to basal levels by lights off (ZT12; Fig. 1B). This peak of plasma 5HT was attenuated following CS exposure (Air vs. CS at ZT6 p<0.05). Further, the peak of plasma 5HT was also advanced by CS exposure (CS, ZT5.03±3.19 vs. Air, ZT7.0±2.26). Together, these data suggest that 3 days of CS exposure affects both the timing (peak phase) and amplitude of the daily rhythm of plasma CORT and 5HT in mice. Further, acute CS had subtle phasic effects on the level of plasma CORT, but more pronounced time-dependent effects were seen in plasma 5HT levels.

**Table 2:** Center of gravity (COG) values for plasma concentration of CORT and 5HT as determined by CircWave analysis in 3 days, 10 days and 6 month air or CS exposed mice.

	Plasma CORT levels		Plasma 5HT levels	
	COG of Air ± SD	COG of CS ± SD	COG of Air ± SD	COG of CS ± SD
**3 days**	9.20±3.07	4.13±3.41	7.00±2.26	5.03±3.19
**10 days**	12.09±2.64**	12.23±2.75	13.57±3.55	8.57±2.64
**6 months**	19.06±3.23	1.37±3.37*	1.33±3.20	19.69±3.50**

Data are shown as mean ± SEM (n = 3–7 per group).

*P<0.05, **P<0.01, significance of rhythmicity as determined by CircWave analysis in air or CS exposed mice.

### Effects of sub-chronic (10 day) CS exposure on daily rhythms of plasma CORT and 5HT

**Figure 2:**
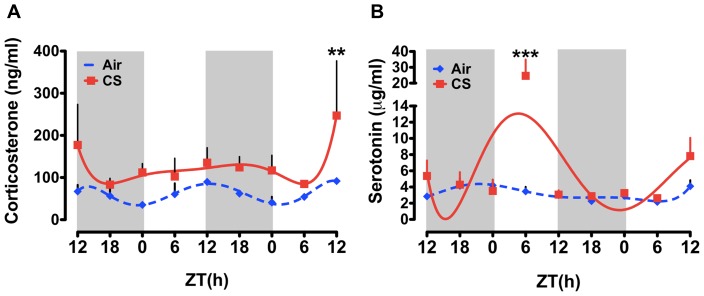
Daily rhythms of plasma CORT and 5HT in mice exposed to sub-chronic (10 day) air or CS. Mice were exposed to CS for 10 days, and plasma samples were collected at 6-h interval for 48 h (starting at ZT12) beginning 24 h after the last exposure. Plasma levels of (A) CORT and (B) 5HT were determined by ELISA. Gray shading indicates the dark phase (ZT12 to ZT24). Data from air- and CS-exposed mice are shown as mean ± SEM (n = 3 mice per group). Data were fit with non-linear regression (multi-order polynomial) analyses. **P<0.01 significant compared to air-exposed mice at ZT12; ***P<0.001 significant compared to air-exposed mice at ZT6.

Given the modest effects of acute CS, we labored to examine the effects of an additional week of CS exposure on plasma CORT and 5HT rhythms. This time frame matches that of our previous study which revealed a significant effect of CS on the level of locomotor activity in mice [39]. As described in methods, mice received 5 h of exposure every day for 10 days. One day after the last exposure, mice were sacrificed beginning at ZT12 every 6 h for 48 h and plasma was collected. As in the 3 d exposure group, we detected a diurnal rhythm of plasma CORT in mice exposed to air for 10 d with a peak at ZT12.09±2.64 (Fig. 2A and Table 2). CS exposed mice had slightly greater plasma CORT levels across the sampling period though a statistically significant difference was only detected at ZT12 on the second day of sampling (Fig. 2A). Sub-chronic CS did not have any effect on the timing of CORT secretion, as the peak in CS plasma was not significantly different from sub-chronic air-exposed mice (air ZT12.09±2.64 vs. CS ZT12.23±2.75; see Table 2).

Overall, 10 d of sub-chronic CS exposure had little effect on plasma levels of 5HT across the 48h-sampling period, with the exception being a considerable increase at ZT6 on the first day of sampling (Fig. 2B). This peak coincides with the approximate time of CS exposure in mice, suggesting a transient after-effect of CS exposure on plasma 5HT. CS did appear to produce a modest phase advance of the rhythm of plasma 5HT such that the peak occurred earlier in CS-exposed mice (ZT8.57±2.64) when compared with air-exposed controls (ZT13.57±3.55; see Table 2).

### Effects of chronic (6 month) CS exposure on daily rhythms of plasma CORT and 5HT

**Figure 3:**
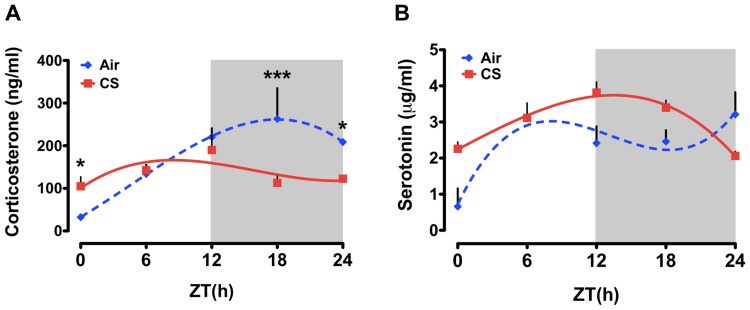
Daily rhythms of plasma CORT and 5HT in mice exposed to chronic (6 month) air or CS. Mice were exposed to chronic CS for 6 months and plasma samples were collected every 6-h interval (ZT0, ZT6, ZT12, ZT18 and ZT24) after 24 h of last CS exposure. Plasma levels of (A) CORT and (B) 5HT and were determined by ELISA. Gray shading indicates the dark phase (ZT12 to ZT24). Data from air- and CS-exposed mice are shown as mean ± SEM (n = 3–4 mice per group). Data were analyzed with non-linear regression (multi-order polynomial) analyses. *P<0.05 significant compared to air-exposed mice at ZT0; *P<0.05 significant compared to CS-exposed mice at ZT24; ***P<0.001 significant compared to CS-exposed mice at ZT18.

Our recent data from acute and sub-chronic CS exposure experiments suggest that CS-induced inflammation is associated with a considerable decline in activity [39], but only modest changes in both the timing and amplitude of CORT and 5HT secretion. Therefore, we sought to determine the timing and amplitude of CORT and 5HT secretion by chronic CS exposure (which leads to the development of COPD/emphysema). As compared to both the acute and sub-chronic air-exposed mice, we detected a robust diurnal rhythm of plasma CORT in chronic air-exposed mice marked by a peak at ZT19.06±3.23 (Fig. 3A and see Table 2). Chronic CS-exposure delayed the peak of CORT secretion (ZT1.37±3.37) and produced phase-specific effects on the overall level of plasma CORT (Fig. 3A).

As in the other air-exposed groups, we detected a diurnal rhythm of plasma 5HT in chronic air-exposed mice with a peak at during the light phase (ZT1.33±3.20; Fig. 3B and see Table 2). However, it is notable that this peak occurred later in younger mice (3 d and 10 d CS exposure peaks at ZT7 and ZT13.4, respectively). Plasma 5HT levels declined across the remainder of the day only to rise again at ZT24 (Fig. 3B). In CS-exposed mice, plasma 5HT levels were elevated relative to air-exposed controls throughout the dark phase (ZT12 and ZT18) but dropped precipitously by the following morning (ZT24). Further, CS appeared to phase delay the timing of peak plasma 5HT levels (Fig. 3B). In air-exposed mice, plasma 5HT peaked near ZT6 while CS exposed mice showed a peak closet to ZT20 (Fig. 3B).

### Putative effects of aging on daily rhythms of plasma CORT and 5HT in mice

**Figure 4:**
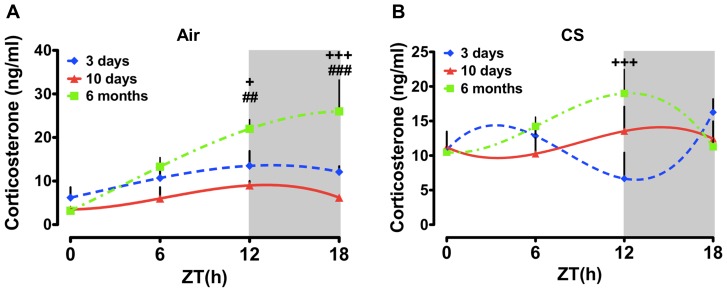
Influence of age on daily rhythms of plasma CORT in air or CS-exposed mice. Adult mice (8–10 weeks old at start of CS exposure) were exposed to air or CS for 3 days (young), 10 days (young) and 6 months (middle aged). Plasma levels of CORT were determined by ELISA. Gray shading indicates the subjective dark phase (ZT12 and ZT18). Data from air- and CS-exposed mice are shown as mean ± SEM (n = 3–7 mice per group). Data were analyzed with non-linear regression (multi-order polynomial) analyses. ^+^ *P*<0.05; ^+ + +^ *P*<0.001 significant compared to 3 days air- or CS-exposed mice; ^# #^ *P*<0.01; ^# # #^ *P*<0.001 significant compared to 10 days air-exposed mice.

**Figure 5:**
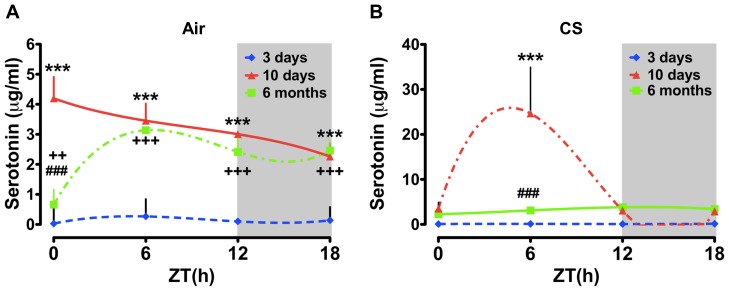
Influence of age on daily rhythms of plasma 5HT in air or CS-exposed mice. Adult mice (8–10 weeks old at start of CS exposure) were exposed to air or CS for 3 days (young), 10 days (young) and 6 months (middle aged). Plasma levels of 5HT were determined by ELISA. Gray shading indicates the dark phase (ZT12 and ZT18). Data from air- and CS-exposed mice are shown as mean ± SEM (n = 3–7 mice per group). Data were analyzed with non-linear regression (multi-order polynomial) analyses. *P<0.05; **P<0.01; ***P<0.001 significant compared to 3 days air- (ZT0–ZT18) or CS- (ZT6) exposed mice; ^+ +^ *P*<0.01; ^+ + +^ *P*<0.001 significant compared to 3 days air-exposed mice (ZT0–ZT18); ^# #^ *P*<0.01; ^# # #^ *P*<0.001 significant compared to 10 days air- (ZT0) or CS- (ZT6) exposed mice.

Given the apparent age-dependent effects on plasma CORT and 5HT rhythms observed in the chronic CS-exposed group, we examined the effects of CS as a function of age/treatment duration across the 24 h day. Our acute and sub-chronic groups were both approximately 2–4 months old at the time of the experiments, whereas our chronic exposure groups were closer to 9–12 months old at the time of euthanasia. As seen in figure 4, CS increased the level of plasma CORT at ZT0 regardless of treatment duration or age, though this effect was most pronounced in the 10 d and chronic CS-exposed groups (Fig. 4A). There was no apparent effect of treatment duration/age on the level of plasma CORT at ZT6 (Fig. 4B). At ZT12 there was a tendency for an increase in CORT levels as a function of age in both the air- and CS-exposed groups (Fig. 4A–B). These data suggest an age-dependent increase in the peak level of CORT in mice. This effect of age was not apparent in the plasma collected at ZT18 (Fig. 4B). Regardless of treatment (CS vs. air), age (3 d, 10 d or 6 mo), or treatment duration plasma 5HT levels were significantly greater in the 10 d and chronic CS-exposed groups (Fig. 5A). Further, there was a tendency for slightly greater plasma 5HT at both ZT12 and ZT18 in the chronic CS-exposed mice (Fig. 5B).

### Plasma levels of CORT and 5HT in non-smokers, smokers, and patients with COPD

**Figure 6:**
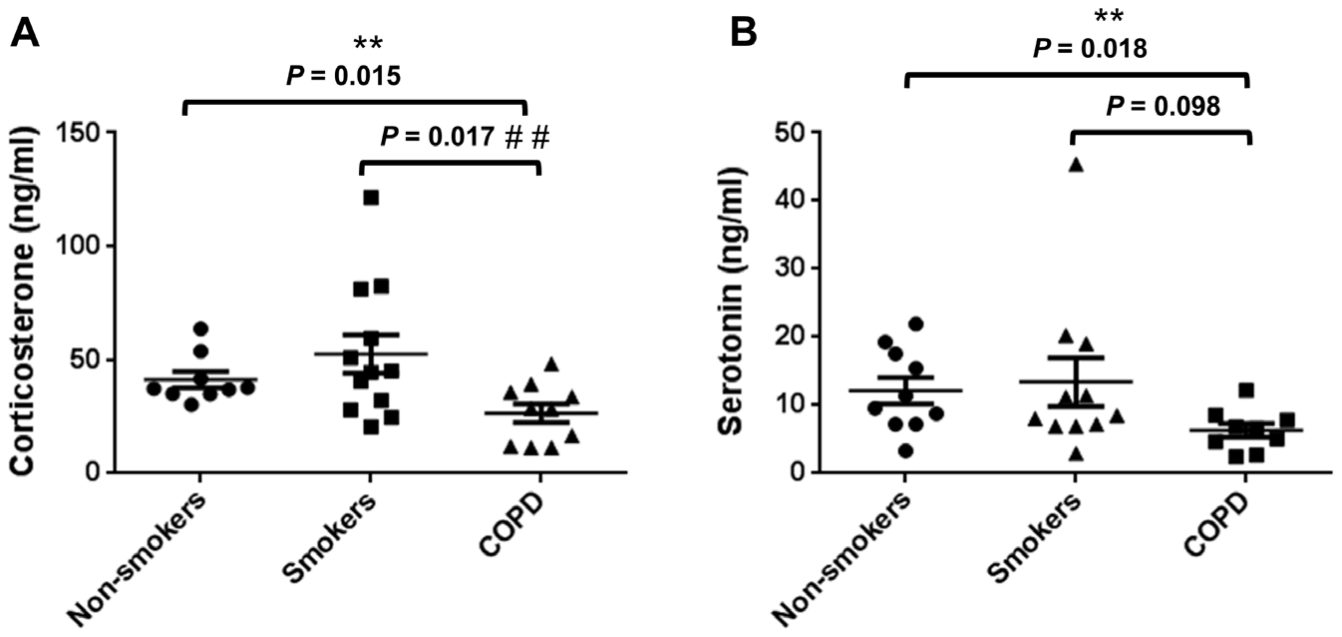
Plasma concentration of CORT and 5HT in non-smokers, smokers and patients with COPD. (A) The level of plasma CORT increased slightly in smokers but was significantly reduced on patients with COPD relative to non-smokers and smokers (current/ex-smokers). (B) COPD patients show lower 5HT concentration than non-smokers and smokers. The level of plasma 5HT was not affected in smokers when compared with non-smokers. Data are shown as mean ± SEM (n = 10–12 subjects per group). **P<0.01 significant when non-smokers were compared to patients with COPD; ^# #^ *P*<0.01 significant when smokers were compared to patients with COPD.

CS can affect physiological rhythms outside the lungs, including rhythmic hormone secretion, and it is not known whether these effects of CS also contribute to the pathophysiology of COPD. Furthermore, since we detected a significant effect of both acute and chronic CS exposure on daily rhythms of plasma CORT and 5HT in mice, we next determined the plasma levels of each hormone in non-smokers, smokers (current/ex-smokers), and COPD patients at approximately the same age (Table 1). Our goal was to determine whether cigarette smoke and COPD have any effect on the secretion of these two stress hormones in humans. We found that the plasma levels of CORT were elevated in smokers when compared with non-smokers (Fig. 6A). These data corroborate with our findings in sub-chronic CS (10 d) exposed mice, wherein CORT levels were increased throughout the 24 h day (see Fig. 2A). In contrast, patients with COPD displayed a significant decline in plasma CORT relative to non-smokers and smokers. These data are in agreement with our results in mice; that is, we observed a significant decline in the level of plasma CORT in chronic CS exposed (COPD model) mice during the active period (night time; see Fig. 3A). We measured comparable effects of smoking and COPD on the level of plasma 5HT. As with CORT, plasma 5HT was slightly elevated in smokers but was significantly reduced in patients with COPD. In contrast with the data from acute CS exposed mice, plasma 5HT level was slightly greater in smokers relative to non-smokers. Interestingly, we did observe an increase in 5HT in mice but the timing of this effect was during the day or inactive period in mice. It is possible the nature of the effect is the same due to the fact that CS exposure occurred during the middle of the light phase (ZT3-9). Thus, the acute effects of CS on serum 5HT were consistent between mice and patients who were smokers (current/ex-smokers).

## Discussion

In mammals, endogenous daily or circadian rhythms of gene expression and physiology depend on the activity of a macromolecular transcription-based oscillator. The circadian oscillator is comprised of several complex and interactive loops of transcription factors, kinases and co-regulators referred to as clock genes [11], [50]. The near 24 h frequency of clock gene expression defines the period of the oscillator and drives circadian rhythms of gene expression, cellular physiology, hormone secretion, metabolism and behavior [50], [51]. In addition to the central circadian pacemaker located in the SCN of the basal hypothalamus, peripheral tissues, including the lung, liver, heart and kidney are comprised of cell-autonomous circadian oscillators [52]–[54]. Clock gene expression in the lungs is altered by CS exposure in both rats and mice [39], [54]–[56]. Circadian or chronodisruption changes mouse lung clock gene expression and lung mechanics [57]–[59]. It is clear from our work and others that disruption of clock function in the lungs can also have a profound influence on pulmonary function and lung pathology [39], [57]–[59]. However, it remains to be determined if CS similarly affects physiological rhythms outside the lungs, including rhythmic hormone secretion, and whether these effects of CS also contribute to the pathophysiology of COPD.

A bevy of evidence supports the notion that cigarette smoking is highly associated with several neuropsychiatric disorders [16], and that the smoking related disease COPD is comorbid with psychological distress [14], [15]. It has been shown that sleep disturbances and night-time symptoms are common in COPD patients [10], suggesting that the function of circadian clock is disrupted in COPD patients [10]–[12], [60]. We have previously determined that CS exposure significantly reduces locomotor activity and alters molecular clock function in the brain and lungs of mice [39]. The impacts of CS exposure on nocturnal activity are particularly profound and suggest the development of a “depression-like” behavioral pattern in these mice [39]. It has been shown that altered rhythms of plasma 5HT, CORT, and cortisol levels and genetic polymorphisms of the clock genes and 5HT-receptors are directly associated with depression, sleep disorders and decreased locomotor activity [23], [38], [46], [49], [61], [62]. Moreover, it has been reported that plasma levels of 5HT and CORT are elevated by CS exposure in mice [63], [64], presumably due to increased systemic oxidative and carbonyl stress [65]. Our aim was to determine if the reduced locomotor activity we have observed following CS exposure [39] had a direct relationship with the effects if CS in the amplitude and timing of plasma 5HT and CORT in mice. In air-exposed mice, plasma 5HT and CORT peaked during the day and night, respectively. Chronic CS-exposure differentially affected both 5HT and CORT levels in a time-dependent manner. Mice exposed to CS showed an increase in plasma 5HT in the early to middle portion of the dark phase. Alternatively, plasma CORT levels dampened overall, with an increase in the early light phase and a reduction during the night. Acute CS-exposure also affected the levels of plasma 5HT and CORT in a time-dependent manner. The level of 5HT surged during the smoke exposure (middle of the day) and then rapidly declined to match the level in air-exposed mice. In contrast, the level of plasma CORT was elevated throughout the day in CS-exposed mice. These shifted and damped rhythms of hormone secretion were associated with repressed activity, particularly during the dark phase [39]. These data suggest that stress induced by CS (both chronic and acute) disrupts rhythmic secretion of plasma CORT and 5HT and that these effects are associated with, and perhaps mechanistically linked to the development of depression in smokers and patients with COPD [39].

Previously, acute and short-term CS exposure in BALB/c mice revealed conflicting influence on CORT secretion [66], [67]. CS exposure for 4 weeks significantly increased plasma CORT [66]. In an independent study of short-term CS exposure plasma CORT levels were not affected [67]. In the present study, acute CS-exposure mice showed plasma CORT peaked at ZT18, and in sub-chronic CS-exposed mice the levels of CORT peaked at ZT12 and remained high throughout all the ZT's in a time-dependent manner in response to CS. The contrasting effects of CS in the acute (3 d), sub-chronic (10 d) and chronic (6 mo) models may be due variation in the phase of exposure (inactive or light phase in 3 d and 6 mo vs. active phase in 10 d), concentration of CS (TPM), main-stream/side-stream delivery method or the duration of CS exposure (2–5 h), which may contribute to the change in the peaking of plasma CORT and 5HT levels observed in the two different acute and sub-chronic models of CS exposure. In another study, the serum levels of CORT remained within the normal range (50–300 ng/ml) when BALB/c and C57BL/6 mice were exposed to sub-chronic CS (600 µg/L) for 5 weeks [68].

The level of 5HT remained unaltered in acute 3 days CS-exposed mice, whereas the plasma 5HT levels peaked at ZT6 in air-exposed mice. Similarly, levels of plasma 5HT peaked at first ZT6 (mid-day) in sub-chronic CS-exposed mice and remain declined in the dark period after exposure in CS-exposed mice. A recent study has demonstrated the involvement of serotonin metabolism in CS-induced oxidative stress in rat lung in vivo [69]. Sprague-Dawley rats were exposed to CS or air for 1 hr daily for 56 days, and 5HT levels were measured in the bronchoalveolar lavage (BAL), lung homogenates and serum [69]. CS exposure did not alter 5HT levels in BAL and lung homogenates. However, serum 5HT levels and MAO-A (monoamine oxidase) activity in the lung were significantly increased in response to CS [69]. These findings indicate that oxidative/carbonyl stress (antioxidant GSH depletion and protein oxidation/carbonylation) in the lung of CS exposed rats correlates with the metabolism of 5HT by MAO-A in the lung. This could lead to enhanced CS-mediated effects, and may play an important role in COPD pathogenesis [69]. Another study has shown a long-lasting effect of smoking in epigenetic regulation of *MAOB* transcription [70]. Both smokers and former smokers as compared to nonsmokers exhibit reduced methylation of *MAOB* promoter, leading to active gene transcription, and hence increased protein concentration of MAO-B as a result of CS-induced increase in nucleic acid demethylase activity [70].

It has been shown that the detrimental metabolic effects of combined long-term CS exposure with high-fat diet in BALB/c mice (CS exposure: 6 days/wk, for 7 wk) did not affect plasma CORT levels in either high-fat diet or CS exposure suggesting that these mice had no additional stress from CS exposure during high fat feeding [71]. Another study on long-term CS exposure in BALB/c mice reported a higher level of CORT in pair-fed (PF) and CS-exposed mice compared to controls [64]. The PF mice showed a significantly higher CORT level compared to CS-exposed mice suggesting the levels of stress imposed by exogenous food restriction (pair feeding) was greater than the voluntary reduction in food intake due to the anorexigenic effect of CS exposure [64]. Recently, a long-term inhalation study with mainstream CS in A/J mice showed a slightly more pronounced increase in plasma CORT levels early at day 72 (∼250 to 340 ng/ml) compared towards the end of the study day 478 (∼160 to 260 ng/ml) [72]. Thus, mainstream CS exposure caused elevated plasma levels of CORT in a mouse model of chronic lung inflammation, tumorigenesis and emphysema [72]. We have shown that chronic CS exposure significantly increased plasma CORT at ZT0, though it remained lower at all-time points (∼100 to 170 ng/ml). This observation may be the result of the lower concentration (∼100 mg/m^3^ TPM) of side-stream CS exposure used, which is minimal compared to the mainstream smoke exposure (∼150–300 mg/m^3^ TPM). Chronic CS exposure did not change plasma levels of 5HT in mice. We speculate that different methods of CS exposure, the strain of mice, concentration of particulates (TPM) time of CS exposure (2–5 h) and the age of mice during exposure may have an impact on the observed plasma levels of CORT following chronic CS. These potential caveats aside, we have for the first time shown a decrease in CORT levels in plasma from patients with COPD compared to smokers and non-smokers. It is not clear whether subjects who were on steroids (smoker/patients with COPD) show any differences in the plasma CORT levels due to lower sample size analyzed in this study. Larger population-based studies will be needed to address the potential influence of steroid treatments on the secretion of circulating CORT levels in smokers and patients with COPD. Further studies on rhythmic regulation of plasma CORT levels in non-smokers, smokers/ex-smokers and patients with COPD may provide better understanding on the role of altered CORT during exacerbations and pathophysiological events in patients with COPD.

Recent studies have shown a positive association between plasma levels of 5HT and 5-HIAA linking severity of depression in patients with COPD implicating their role in the development of COPD [23], [24]. A significant correlation with increased plasma levels of 5HT in patient with COPD associated with increasing age has been shown in a cross-sectional study [24]. Another recent study has demonstrated that the serotonin metabolites as possible biomarkers for depression in patients with COPD and found association of increased plasma 5HIAA level in depressed COPD patients [23]. Plasma levels of 5HT among the four subject groups (nonsmokers, smokers, non-depressed COPD, and depressed COPD patients) were not different [23]. Our data on plasma levels of 5HT in COPD have trend to be low compared to non-smoker s and smokers, but are not significant which is in contrast to the earlier report. These contrasting observations on the plasma levels of 5HT in COPD patients may be due to other confounding factors, such as serotonin transporter gene polymorphism and pulmonary hypertension [73] associated with COPD patients or other comorbidities of COPD including pulmonary artery pressure or vascular dementia [74]. Future studies will provide better insights on the confounding factors and their possible role in altered plasma levels of 5HT in patients with COPD. Overall, these data reveal that CS-induced disruption of activity, an indirect marker of neurophysiological disease, is associated with altered daily rhythms of plasma CORT and 5HT. Furthermore, the disrupted rhythms of CORT and/or 5HT may be a critical factor in the appearance of depressive behavior following CS-exposure. These findings may have a larger impact in cigarette smoke-induced chronic lung diseases, such as COPD and associated comorbidities where depression is a common phenomenon, including addiction, predisposition to cancer and other mental health disorders.

In conclusion, we found that CS, which we have previously shown produces depression-like behavior, lung oxidative/carbonyl stress and inflammation, emphysema and altered clock gene expression in the brain and lung, also affects the timing and amplitude of plasma 5HT and CORT secretion. The locus of these effects remains to be determined. It is possible, given the effects of CS on clock gene expression rhythms in the brain and lungs, that it has similar effects on the timing of clock gene expression in the adrenal gland, entero-chromaffin cells and the neuroendocrine pulmonary cells releasing 5HT in the lungs. It is also possible that CS alters the timing of 5HT release from neurons in the raphe nuclei. It remains to be seen of the effects of CS on mood can be directly and mechanistically linked to altered clock function in these areas. Together, these data reveal a significant impact of CS exposure and COPD on the timing and amplitude of hormone secretion that could explain some of the detrimental effects of CS on cognitive function, mood/anxiety and sleep quality in patients with COPD.
